# New clinical algorithm including fungal biomarkers to better diagnose probable invasive pulmonary aspergillosis in ICU

**DOI:** 10.1186/s13613-021-00827-3

**Published:** 2021-03-08

**Authors:** Joffrey Hamam, Jean-Christophe Navellou, Anne-Pauline Bellanger, Stéphane Bretagne, Hadrien Winiszewski, Emeline Scherer, Gael Piton, Laurence Millon, Collaborative RESSIF group

**Affiliations:** 1 Medical Intensive Care Unit, University Hospital of Besançon, 25000 Besancon, France; 2 Intensive Care Unit, Libourne Hospital, 33500 Libourne, France; 3 Parasitology-Mycology Department, University Hospital of Besançon, 25000 Besançon, France; 4 UMR 6249 CNRS Chrono-Environnement, University of Bourgogne Franche-Comté, 25000 Besançon, France; 5 Institut Pasteur, CNRS, Unité de Mycologie Moléculaire, Centre National de Référence Mycoses Invasives et Antifongiques, UMR 2000, Paris, France; 6 Université de Paris, Laboratoire de Parasitologie-Mycologie, Hôpital Saint Louis, AP-HP, Paris, France

**Keywords:** Invasive aspergillosis, Intensive care unit, Clinical algorithm, Fungal biomarkers, Galactomannan antigen, *Aspergillus* qPCR

## Abstract

**Background:**

The classification of invasive pulmonary aspergillosis (IPA) issued by the European Organization for the Research and Treatment of Cancer/Mycoses Study Group Education and Research Consortium (EORTC/MSGERC) is used for immunocompromised patients. An alternative algorithm adapted to the intensive care unit (ICU) population has been proposed (AspICU), but this algorithm did not include microbial biomarkers such as the galactomannan antigen and the *Aspergillus* quantitative PCR. The objective of the present pilot study was to evaluate a new algorithm that includes fungal biomarkers (BM-AspICU) for the diagnosis of probable IPA in an ICU population.

**Patients and methods:**

Data from 35 patients with pathology-proven IPA according to European Organization for the Research and Treatment of Cancer/Mycosis Study Group (EORTC/MSGERC)-2008 criteria were extracted from the French multicenter database of the Invasive Fungal Infections Surveillance Network (RESSIF). The patients were investigated according to the AspICU algorithm, and the BM-AspICU algorithm in analyzing the clinical, imaging, and biomarker data available in the records, without taking into account the pathology findings.

**Results:**

Eight patients had to be excluded because no imaging data were recorded in the database. Among the 27 proven IPAs with complete data, 16 would have been considered as putative IPA with the AspICU algorithm and 24 would have been considered as probable IPA using the new algorithm BM-AspICU. Seven out of the 8 patients with probable BM-AspICU IPA (and not classified with the AspICU algorithm) had no host factors and no *Aspergillus*-positive broncho-alveolar lavage fluid (BALF) culture. Three patients were non-classifiable with any of the two algorithms, because they did not have any microbial criteria during the course of the infection, and diagnosis of proven aspergillosis was done using autopsy samples.

**Conclusion:**

Inclusion of biomarkers could be effective to identify probable IPA in the ICU population. A prospective study is needed to validate the routine application of the BM-AspICU algorithm in the ICU population.

## Background

The diagnosis of invasive pulmonary aspergillosis (IPA) in intensive care unit (ICU) remains a challenge. Definitions of invasive fungal diseases were proposed in 2002, then updated in 2008 and in 2019, by a consensus group of the European Organization for Research and Treatment of Cancer (EORTC) and the Mycoses Study Group Education and Research Consortium (MSGERC) [1, 2]. The EORTC/MSGERC classification is not suitable for ICU population, as immunocompetent patients admitted to the ICU for severe acute illness, while at risk for IPA, do not have the host factors described in the EORTC/MSGERC definitions [1]. This EORTC/MSGERC definitions were first created in order to homogenize immunocompromised population included in clinical trials. Proven cases were defined by positive histological examination with visible hyphae or positive culture on sterile material. Possible cases were defined by the presence of host factors and radiological criteria, probable cases were defined by host factors, radiological and microbiological criteria (culture, galactomannan (GM) antigen) [1]. *Aspergillus* quantitative polymerase chain reaction (qPCR) has been included as microbiological criterion in the 2019 update [2].

Because data are lacking for IPA diagnosis in ICU population, an alternative clinical algorithm, AspICU, more adapted to critically ill patients, was validated by a prospective multicenter study [3]. The objective of the AspICU algorithm aimed at discriminating *Aspergillus* colonized patients from patients with a probable IPA. In order to avoid confusion with the “probable” term described in the EORTC/MSGERC-2008, the term used in the AspICU algorithm was “putative”. Recently, new case definitions have been proposed for influenza-associated invasive aspergillosis (IAPA) and Covid-19-associated invasive aspergillosis (CAPA), which include fungal culture and biomarkers as requirement for putative/probable cases [4–6].

Unlike the EORTC/MSGERC classification, the AspICU algorithm used clinical signs, less restrictive host factors, and *Aspergillus-*positive culture from respiratory tract to define “putative” aspergillosis. However, the AspICU algorithm did not use the GM antigen detection because it was shown to be less reliable in non-neutropenic patients [7]. Moreover, the AspICU classification did not consider the detection of *Aspergillus* DNA using qPCR in blood samples or broncho-alveolar lavage fluid (BALF) for the diagnosis of IPA.

The EORTC/MSGERC classifications are used to enroll patients into clinical trials/diagnostic evaluations and not to direct or guide patient care. By contrast, the AspICU algorithm was developed to discriminate colonization from probable IPA in ICU patient with *Aspergillus*-positive endotracheal aspirate culture and help in therapeutic decision-making.

We hypothesized that the strategy to diagnose probable IPA in the ICU population could be improved, so that the patients could be treated earlier, especially if they do not have immunosuppression criteria. We propose here a new algorithm, entitled BM-AspICU, based on our experience and on the literature, mixing both EORTC/MSGERC and AspICU criteria and including fungal biomarkers, such as the GM antigen and the *Aspergillus* qPCR [8].

The objective of this pilot study was to evaluate mycology assay positivity that would allow different classifications in the absence of a proven diagnosis. The patients with proven cases collected by the French Invasive Fungal Infections Surveillance (RESSIF) network were investigated according to the AspICU algorithm, and the BM-AspICU algorithm in analyzing the clinical, imaging, and biomarker data available in the records, without taking into account the pathology findings.

## Methods

### Collection of EORTC/MSGERC-proven IA cases

The RESSIF network was launched in 2012 by the National Reference Center of Invasive Mycoses and Antifungals to collect cases of invasive fungal infections associating microbiological and clinical data. The RESSIF network includes 29 collaborating centers who declare the proven and probable cases according to EORTC/MSGERC-2008. For the present study, only the proven IPAs occurring in ICU were considered for homogenization purpose and also because the diagnosis of aspergillosis was undisputable. Indeed, ICU patients do not generally have host factors necessary for defining probable IPA, and are therefore not recorded in the network unless they present host factors, which create biases with ICU patients without host factors. Moreover, the diagnosis of probable cases often relies on biomarkers and not on culture, which would have interfered with the present evaluation of the added value of biomarkers. Therefore, probable cases were not considered.

The analysis of the aspergillosis records between January 2012 and December 2017 retrieved 35 patients over 18 years old with proven IPA and admitted to the ICU. Additional data were obtained after the analysis of anonymized hospitalization records of the 35 patients to create the BM-AspICU database. Radiological data were analyzed from hospitalization records. The items collected and taken into account for each algorithm are listed in Table 1.

**Table 1:** Diagnostic criteria for invasive pulmonary aspergillosis according to EORTC/MSGERC-2008, EORTC/MSGERC-2019, AspICU and BM-AspICU

Criteria	EORTC/MSGERC-2008	EORTC/MSGERC-2019	AspICU	BM-AspICU
	Host risk factors (immunosuppression)			
Neutropenia (< 500 neutrophils/mm^3^ for > 10 days)	X	X	X	X
Receipt of an allogenic stem cell transplant	X	X	X	X
Corticosteroids > 0.3 mg/kg/day for > 3 weeks	X	X	X	X
Treatment with recognized T-cell immunosuppressant for more than 90 days	X	X	X	X
Inherited severe deficiency	X	X	X	X
Underlying hematological or oncological malignancy treated with cytotoxic agents	X	X	X	X
Ibrutinib treatment		X	X	X
	Other risk factors			
Chronic obstructive pulmonary disease			X	X
Viral respiratory diseases (influenza infection, SARS-CoV2 infection, etc.)			X	X
Cirrhosis, hepatic insufficiency			X	X
Other (diabetes, chronic alcohol abuse, chronic diseases, cardiac surgery, etc.)			X	X
	Clinical features			
Fever refractory to > 3 days of antibiotherapy			X	X
Pleuritic chest pain			X	X
Dyspnea			X	X
Hemoptysis			X	X
Respiratory insufficiency despite ventilation support			X	X
	Imaging			
CT scan of the lung	X	X	X	X
Chest X-ray			X	X
Air-crescent sign	X	X	X	X
Cavity	X	X	X	X
Dense, well-circumscribed lesion(s) with or without halo sign	X	X	X	X
Diffuse reticular and alveolar opacities		X	X	X
Nonspecific infiltrates and consolidation		X	X	X
Pleural fluid			X	X
Wedge-shaped infiltrate		X	X	X
Tree-in-bud pattern			X	X
	Mycological culture			
Positive direct examination showing hyphae	X	X	X	X
Positive *Aspergillus* culture in BALF	X	X	X	X
Positive *Aspergillus* culture in lower respiratory tract specimen	X	X	X	X
	Fungal biomarkers			
BALF galactomannan	X	X		X
BALF *Aspergillus* qPCR		X*		X
Serum/plasma galactomannan	X	X		X
Serum/plasma *Aspergillus* qPCR		X*		X

^*^ Two consecutive qPCR tests positive in blood, or one qPCR test positive in blood and one qPCR test positive in BALF

The RESSIF network was approved by the Institut Pasteur institutional review board (IRB #2009-34). Approval of the "Commission Nationale de l'Informatique et des Libertés" was obtained, ensuring that patient’s data were kept and used according to French regulation. The BM-AspICU substudy was approved by the coordinating committee of RESSIF in April 2019. All patients’ medical data analyzed in this study were anonymized.

### Classification using the different algorithms

All patients with proven IPA were classified according to the AspICU algorithm and the BM-AspICU algorithm, without taking into account the pathology findings.

The AspICU algorithm aimed at discriminating *Aspergillus* colonized patients from patients with a putative IPA. Putative cases were defined by positive *Aspergillus* culture in lower respiratory tract, host factors (neutropenia, underlying hematological or oncological malignancy treated with cytotoxic agents, glucocorticoid treatment (> 20 mg/day), congenital or acquired immunodeficiency), clinical, and radiological criteria; a second mycological criterion (semiquantitative *Aspergillus*-positive culture of BALF (+ or + +) with a positive direct examination showing branching hyphae was necessary if host risk factor was lacking [3].

**Fig. 1:**
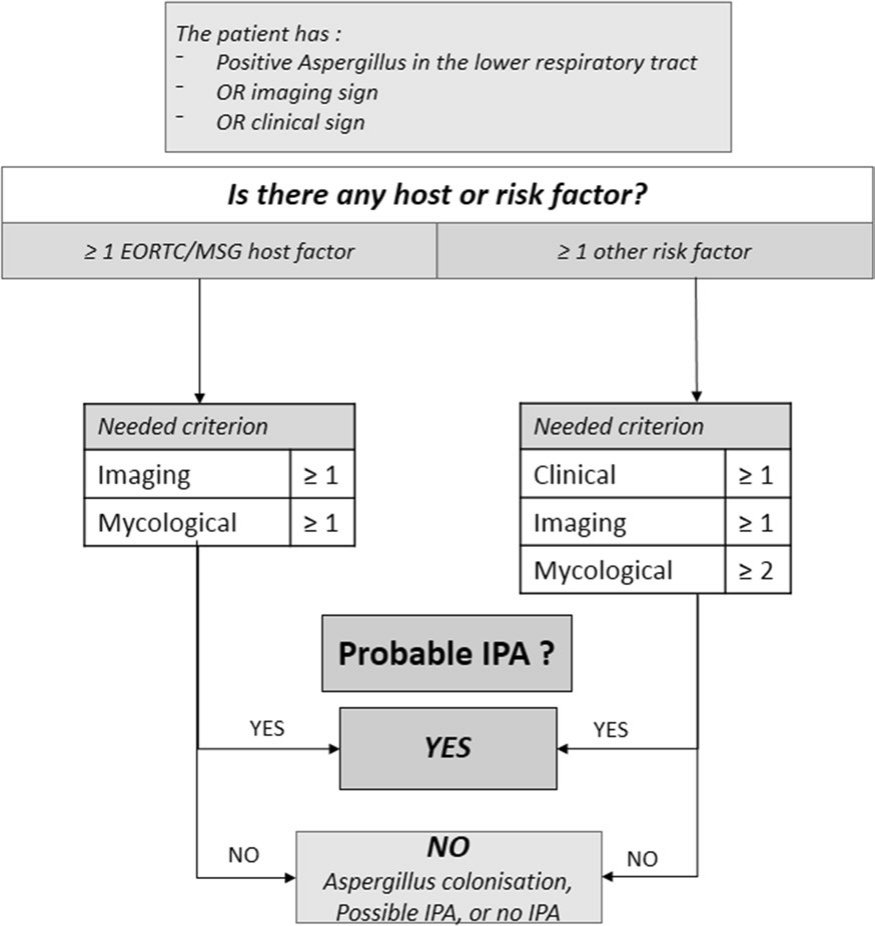
BM-AspICU algorithm to discriminate probable invasive pulmonary aspergillosis (IPA)

The new BM-AspICU algorithm proposed in this study was based on our experience and data from literature (Fig. 1). In the BM-AspICU algorithm, we take into account fungal biomarkers such as the GM antigen and the *Aspergillus* qPCR for the classification. Risk factors were not considered as entry criteria, and BM-AspICU has been designed to be applied to any patient requiring ICU admission for respiratory distress, regardless of risk factors.

The entry criterion could be either a positive *A. fumigatus* culture in the lower respiratory tract, or imaging signs, or a clinical sign (respiratory worsening, or fever after antibiotics ≥ 3 days) (Fig. 1). Then, the second step was to look for any host factor, including those described in the EORTC/MSGERC-2008 classification, and other risk factors as listed in Table 1. If the patient had any EORTC/MSGERC-2008 host factor, only one radiological criterion and one mycological criterion were needed to categorize the patient as probable IPA. On the other hand, if the patient did not have any host factor according to the EORTC/MSGERC-2008 criteria, but presented at least one other risk factor, one clinical criterion, one radiological criterion and two mycological criteria (including GM antigen and *Aspergillus* qPCR in serum and BALF) were needed to categorize the patient as probable IPA.

## Results

Data for 35 patients with EORTC/MSGERC-proven IPA and having been hospitalized in ICU were analyzed. The patients had a median age of 59 years [25–72] and were mostly men (74%). Among these 35 proven IPAs, eight had to be excluded because imaging was not available (not done or not described in details in the medical file). Among the 27 EORTC/MSGERC-2008 proven IPA patients included, 11 had an EORTC/MSGERC-2008 host factor: five solid organ transplant, and 6 hematological diseases (Table 2). The 16 other patients had other condition such as chronic alcoholism (6), active smoking (4), chronic obstructive pulmonary disease (3), diabetes (3), cardiac surgery (1), Basedow disease (1), Still’s disease (1), gout attack (1), massive exposure to demolition work (1), idiopathic pulmonary fibrosis (1), vasculitis (1), drowning in mud while in alcohol-induced coma (1). Several of these conditions were cumulative for some patients (8 patients with at least two risk factors).

**Table 2:** Details of host factors, imaging, and fungal criteria for each patient. Classification as putative or probable invasive pulmonary aspergillosis was done according to AspICU algorithm, as previously published (3), and according the BM-AspICU algorithm proposed in this study

Patients with EORTC/MSGERC-2008 host factor												
	Age	Sex	EORCT/MSGERC-2008 host factor	EORTC/MSGERC-2008 imaging	*Aspergillus*-positive culture	GM serum	GM BALF	Outcome 3 months	AspICU	BM-AspICU	Mycological criteria IAPA	Mycological criteria CAPA
P1	66	M	AML	Condensation	None	Pos* (0.98)	Not done	alive	Not sortable	Probable	Yes	Yes
P2	25	M	SOT (kidney)	Micronodules	BALF	Pos* (1.05)	Not done	dead	Putative (4a + 4b)	Probable	Yes	Yes
P3	48	F	SOT (heart)	Nodules, condensations	Tracheal aspirate	Not done	Not done	dead	Putative (4a)	Probable	No	No
P4	58	F	SOT (lung)	Opacities	Bronchial and pleural aspirates	Neg	Neg	alive	Putative (4a)	Probable	No	No
P5	43	M	Lymphoma	Micronodules, ground glass opacities	BALF	Neg	Neg*	alive	Putative (4a + 4b)	Probable	Yes	Yes
P6	63	F	SOT (liver)	Nodules	BALF	Pos* (2.3)	Pos* (0.88)	dead	Putative (4a + 4b)	Probable	Yes	Yes
P7	49	M	Leukemia	Nodules, condensations	Bronchial aspirate	Pos (4)	Pos (4)	dead	Putative (4a)	Probable	Yes	Yes
P8	65	M	SOT (heart)	Condensation, ground glass opacities	BALF tracheal aspirate	Pos (1.7)	Neg	dead	Putative (4a + 4b)	Probable	Yes	Yes
P9	65	F	Lymphoma	ground glass opacities	Bronchial aspirate	Neg	Not done	alive	Putative (4a)	Probable	No	No
P10	59	M	AML	Condensation, ground glass opacities	None	Neg	Not done	dead	Not sortable	Not sortable	No	No
P11	56	M	AML	Nodules	None	Not done	Not done	dead	Not sortable	Not sortable	No	No

Patients without EORTC/MSG-2008 host factor												
	Age	Sex	Risk factors	Imaging	*Aspergillus-* positive culture	GM serum	GM BALF	Outcome 3 months	AspICU	BM-AspICU	IAPA criteria	CAPA criteria
P12	58	M	Chronic alcoholism and massive exposure during demolition work	Widespread opacities	BALF, tracheal aspirate	Pos (3.46)	Not done	alive	Putative (4b)	Probable	Yes	Yes
P13	50	F	Basedow and toxic agranulocytosis	Condensations	BALF, tracheal aspirate	Not done	Not done	dead	Putative (4b)	Probable	Yes	Yes
P14	72	F	Diabetes	Condensations and ground glass opacities	BALF, tracheal aspirate	Not done	Not done	dead	Putative (4b)	Probable	Yes	Yes
P15	62	M	Cardiac surgery	Nodular and cavitary lesions	BALF, tracheal aspirate	Neg	Not done	dead	Putative (4b)	Probable	Yes	Yes
P16	25	M	Still’s disease and steroid	Pleural effusion	BALF	Pos (1.8)	Negative	dead	Putative (4b)	Probable	Yes	Yes
P17	68	M	Gout attack, steroid	ground glass opacities	BALF, tracheal aspirate	Pos (1.61)	Not done	dead	Putative (4b)	Probable	Yes	Yes
P18	40	M	Chronic alcoholism and diabetes	Multiple nodules	BALF	Pos (1.07)	Pos (2.66)	dead	Putative (4b)	Probable	Yes	Yes
P19	70	F	Lung cancer	Nodules	BALF, tracheal aspirate	Pos (1.06)	Pos (9.5)	dead	Putative (4b)	Probable	Yes	Yes
P20	75	M	COPD, adenocarcinoma	Nodules	2 bronchial aspirates	Neg	Neg	dead	Not sortable	Probable	No	No
P21	57	F	Chronic alcoholism and active smoker	Micronodules and condensation	Tracheal and bronchial aspirates	Pos (1.27)	Not done	alive	Not sortable	Probable	Yes	Yes
P22	58	F	Steroid, vasculitis	Opacities	Tracheal aspirate	Pos (5)	Not done	dead	Not sortable	Probable	Yes	Yes
P23	64	M	Diabetes, active smoking, drowning in mud while in alcohol-induced coma	Diffused opacities, halo sign	Tracheal aspirate	Pos (3)	Not done	dead	Not sortable	Probable	Yes	Yes
P24	70	M	Chronic alcoholism and COPD	Abscesses	Tracheal aspirate Pericardial fluid	Not done	Not done	dead	Not sortable	Probable	No	No
P25	69	M	Chronic alcoholism and COPD and active smoker	Nodule, cavity	Pleural fluid	Pos (2.5)	Not done	alive	Not sortable	Probable	Yes	Yes
P26	62	M	Idiopathic pulmonary fibrosis	Ground glass opacities	None	Neg	Not done	dead	Not sortable	Not sortable	No	No
P27	40	M	Chronic alcoholism and Protein S deficiency and active smoker	Condensations	Bronchial aspiration	Pos (1.34)	Pos (3.6)	dead	Not sortable	Probable	Yes	Yes

Mycological criteria proposed in the new case definitions of putative Influenza-associated pulmonary aspergillosis (IAPA) [5] and putative Covid-19 associated pulmonary aspergillosis (CAPA) [6] were also assessed in our series

Requirement for putative IAPA are radiological (any infiltrate) associated to positive culture from BALF, positive GM in BALF (≥ 1.0) or positive GM in serum (≥ 0.5)

Requirement for putative CAPA are nonspecific radiology signs associated with two or more positives across different test types or multiple positives within one test type, from the following (positive culture from BALF, positive GM in BALF (≥ 1.0), positive GM in serum (≥ 0.5), positive qPCR in BALF or blood, positive beta-D glucan in serum/plasma. In case of radiology typical of IA, one positive mycological tests as listed above is sufficient

4a and 4b refer to criteria 4 from AspICU algorithm, detailed in reference [3]

4a: host risk factors according to AspICU algorithm: one of the following condition (neutropenia; underlying hematological or oncological malignancy treated with cytotoxic agents; glucocorticoid treatment; congenital or acquired immunodeficiency) as described in reference [3]

4b: semiquantitative *Aspergillus*-positive culture of broncho-alveolar lavage fluid (+ or + +), with a positive cytological smear showing branching hyphae

^*^ Positive qPCR on the same sample

The main clinical sign suggestive of IPA was adverse respiratory outcome after antibiotic therapy and involved 23 patients. Overall 90-day mortality rate was 74% in this series. It was higher in non-immunocompromised patients (13/16 [81%]) than in immunocompromised patients with EORTC/MSGERC-2008 host criteria (7/11 [63%]), but difference was not significant (Fischer test *p* > 0.05).

Among the 27 EORTC/MSGERC-2008-proven IPA patients included, 13 had an EORTC/MSGERC-2008 imaging sign, mostly nodules and micronodules (Table 2). The 14 other patients had other, less specific imaging signs, such as condensations (6), ground glass opacities (7), abscesses (4), opacity (3), and pleural effusion (1) (most of these imaging signs are now included in the EORTC/MSGERC-2019).

Among the 27 proven IPA patients included, 4 patients did not have any *Aspergillus*-positive culture from lower respiratory tract specimen or BALF during the monitoring of patient (P1, P10, P11, P26). Patient P1 had a positive GM and was identifiable as probable only with BM-AspICU. The 3 other patients were non-classifiable with any of the two algorithms, because they did not have any microbial criteria (negative mycological culture, negative biomarker or absence of sampling) during the course of the infection, and diagnosis of proven aspergillosis was done using autopsy samples.

Among the 23 other patients, 20 patients had at least one *Aspergillus*-positive culture in respiratory tract (5 in BALF only, and 8 in other lower respiratory tract specimens only such as tracheal or bronchial aspirate, and 7 in both BALF and other respiratory samples). Three patients had other samples with positive *Aspergillus* culture (2 pleural fluids, one pericardial fluid). The strain identified was *A. fumigatus* in 20/22 of patients, *Aspergillus (Emericella) nidulans* was identified in one patient, and *Aspergillus flavus* in another patient.

Among the 27 proven IPA patients included, 15 had a positive GM in serum (median 1.7 [0.98–5]), 4 of them also positive in BALF (median 3.33 [0.88–9.5]), and only four had a positive *Aspergillus* qPCR (3 in serum and one in BALF). The *Aspergillus* qPCR was not systematically performed (only 13/27 patients had at least one serum or BALF tested for *Aspergillus* qPCR).

All in all, out of the 27 patients analyzed, 16 would have been considered as putative IPA following the AspICU algorithm: 8 patients with host risk factors (“4a” criterion), and 8 patients without host risk factors, but fulfilling “4b” criterion (*Aspergillus-*positive culture of BALF with direct examination of hyphae) (Table 2). Otherwise, 24 patients would have been considered as probable IPA following the BM-AspICU algorithm. Among the 8 patients that have been identified as probable IPA using the BM-AspICU algorithm, but not by the AspICU, there were one patient (P1) with host factor, a serum-positive GM and a serum-positive *Aspergillus q*PCR; and 7 patients (P20, P21, P22; P23, P24, P25, P27) without host factors or positive BALF culture, but with at least 2 other positive mycological results (at least 2 positive cultures (other than BALF), or one positive culture and at least one positive GM in serum or BALF) (Table 2).

Mycological criteria required for putative IAPA include positive culture from BALF, positive GM in BALF (≥ 1.0) and positive GM in serum (> 0.5) [5]; in the absence of specific radiologic sign, mycological criteria required for putative CAPA are 2 or more positives across different test types or multiple positives within one test type, from the following: positive culture from BALF, positive GM in BALF (≥ 1.0), positive GM in serum (≥ 0.5), positive qPCR in BALF or blood, positive beta-D glucan in serum/plasma [6]. When applying these criteria in our series, 19 patients could have been considered as probable IPA. The five additional patients identified as probable by BM-AspICU (P3, P4, P9, P20, P24) had positive culture in tracheal or bronchial aspirates (which were not taken into account in the CAPA/IAPA definitions).

## Discussion

The present study showed that the new algorithm BM-AspICU by adding *Aspergillus* qPCR and GM antigen detection in the diagnostic strategy of IPA in the ICU population allowed to identify more patients with probable BM-AspICU IPA (*n* = 24) compared to putative IPAs of the AspICU algorithm (*n* = 16).

The AspICU algorithm did not include any fungal biomarkers. In the absence of host risk factors (immunosuppression), *Aspergillus*-positive culture of BALF with direct examination of hyphae is the only mycological criterion to classify the case as putative aspergillosis [3]. In our study, 16 patients did not have any host risk factor, only 8 of them had a positive BALF and could be classified as putative aspergillosis using the AspICU classification. With the BM-AspICU algorithm, we could identify 8 additional patients as “probable” IPA when considering positive GM, positive *Aspergillus* qPCR in serum and/or BALF or another positive culture of any type of samples, as a second mycological criterion. The inclusion of these biomarkers is in agreement with recent recommendations from the European Society for Clinical Microbiology and Infectious Diseases, the European Confederation of Medical Mycology and the European Respiratory Society (ESCMID-ECMM-ERS joint guidelines) [8] and the American Thoracic Society [9]. The *Aspergillus* qPCR was not recognized as microbiological criterion by the EORTC/MSGERC-2008 classification [1], which could explain the low number of cases for which the analysis was performed in this retrospective study. In the meantime, revisions of the EORTC/MSGERC criteria were published in December 2019 and now, two consecutive positive PCR in blood, or one PCR test positive in blood and one PCR positive in BALF, are considered as mycological criteria for probable IPA [2]. Performance of GM and *Aspergillus* PCR in BALF and serum were also evaluated in COVID-19-associated pulmonary aspergillosis [10–12], and *Aspergillus* qPCR was taken into account in recent definition cases of CAPA [6].

Some similarities between BM-AspICU and CAPA definitions can be noted: 1) considering all biomarkers (GM and *Aspergillus* qPCR) in serum and BALF as mycological criteria and 2) varying the number of required mycological positive tests according to the type of patients. There are also main differences: 1) in the BM-AspICU, we considered positive culture from any respiratory specimens (including tracheal and bronchial aspirate), and not only from BALF or  non-directed bronchial lavage, and 2) in the BM-AspICU, we proposed to outweigh the lack of host factor by the number of mycological criteria (EORTC/MSGERC host factor: only one mycological criteria; no host factor: ≥ 2 mycological criteria) while in CAPA definitions, the lack of specific radiology is outweighed by the number of mycological criteria (radiology typical of IA: only one mycological criteria; nonspecific radiology: ≥ 2 mycological criteria).

The EORTC/MSGERC-2008 classification relied mostly upon host factors and specific imaging signs. This diagnostic approach is however insufficient in the ICU where symptoms such as persistent fever, fever recrudescence under antibiotic, chest pain or acute respiratory distress syndrome seem essential to evoke an IPA. In our study, 16/27 (59%) patients had positive *Aspergillus*-positive culture in lower respiratory tract specimen (tracheal or bronchial aspirate) and all the patients had at least one clinical sign evoking an IPA.

Given the increasing evidence that radiological manifestations are more varied than previously described, and the greater number of abnormalities that could be seen thanks to the new imaging techniques, imaging criteria for probable IPA were expanded to include wedge-shaped and segmental or lobular consolidation in the revised EORTC/MSGERC-2019 classification [2]. In the BM-AspICU algorithm, we propose to use broader criteria, as proposed in the AspICU algorithm [3]. Indeed, in our study diffuse or ground glass opacities were the only radiological feature found in 6 out of the 27 patients with proven IPA.

The absence of EORTC/MSGERC-2008 host criteria in 60% (16/27) patients had probably contributed to delayed diagnosis and delayed treatment, which explains in part the very high mortality rate in this series. Therefore, to resume our strategy, in the ICU, patients with respiratory worsening, fever refractory to antibiotic therapy, and a first *Aspergillus*-positive culture in tracheal or bronchial aspirate or a first positive fungal biomarker, should first be considered for EORTC/MSGERC-2019 host risk factors. If EORTC/MSGERC-2019 host risk factors are identified, no additional mycological sign is needed to immediately start antifungal treatment and continue fungal monitoring. If the patient presents other risk factors, as listed in Table 1, an active fungal surveillance should be triggered (culture of respiratory tract specimens, GM and *Aspergillus* qPCR in serum or BALF) and as soon as a second mycological argument is obtained (positive GM, positive *Aspergillus* qPCR, positive *A. fumigatus* culture), the patient should benefit from an antifungal treatment.

We acknowledge several limitations to our study. First, the retrospective design of the study, with collection of data from the RESSIF network: therefore, some relevant data to describe IPA cases in ICU patients, such as duration of mechanical ventilation before IPA, severity scores, organ dysfunctions were not recorded and could not be provided. Second, the low number of proven IPA obtained from the RESSIF database: 35 recorded between 2012 and 2017. However, in the absence of autopsy to ascertain the diagnosis, the more reliable criterion to stay homogeneous was to consider only proven cases. In doing so, we probably increase the number of patients with advanced disease, and therefore, more prone to present positive biomarkers. Third, we were not able to obtained systematic reliable timing of the positivity of the biomarkers and the culture compared to the date of the positive biopsy. To know these elements could have an interesting clinical impact for initiating a specific treatment without waiting for a pathology confirmation. Fourth, we were not able to perform centralized reading of the imaging data and relied on the conclusion made by different radiologists, which introduce biases in the interpretation. At last, we did not have a control group to evaluate the specificity of the BM-AspICU algorithm. However, false-positive biomarker results are always difficult to assess given the difficulty to exclude the diagnosis of invasive aspergillosis. The benefit/risk balance for the patient is in favor of over diagnosing and treating a patient wrongly rather than underdiagnosing patients with IPA.

## Conclusions

Strict interpretation of the host factors for invasive fungal infection has contributed in some instances to missed diagnosis of IPA in ICU [3, 13]. We therefore think the ICU patients should be considered at risk of IPA independently of their immunity status. Since early IPA diagnosis remains a challenge, biomarkers should be integrated to consider as many patients as possible to improve the prognosis. Including biomarkers may help in decision-making to start antifungal treatment in ICU patients with hematological malignancies, but also in ICU patients with other risk factors. The BM-AspICU algorithm was based on retrospective analysis of the RESSIF database and needs to be validated on a prospective study, to determine if fungal biomarkers, such as GM antigen detection and *Aspergillus* qPCR, in ICU patients without EORTC/MSGERC-2019 host factors, should be systematically part of the IPA diagnostic strategy. In the future, the BM-AspICU algorithm should be assessed for CAPA and IAPA.

## Data Availability

The datasets used and/or analyzed during the current study are available from the corresponding author on reasonable request.

## Abbreviations

BALF: Broncho-alveolar lavage fluid
BM: Biomarker
CAPA: Covid-19-associated invasive aspergillosis
EORTC: European Organization for Research and Treatment of Cancer
MSGERC: Mycoses Study Group Education and Research Consortium
GM: Galactomannan
ICU: Intensive care unit
IPA: Invasive pulmonary aspergillosis
IAPA: Influenza-associated invasive aspergillosis
qPCR: Quantitative PCR
RESSIF: Réseau de Surveillance des Infections Fongiques
